# Nematicidal Bacteria Associated to Pinewood Nematode Produce Extracellular Proteases 

**DOI:** 10.1371/journal.pone.0079705

**Published:** 2013-11-07

**Authors:** Gabriel Paiva, Diogo Neves Proença, Romeu Francisco, Paula Verissimo, Susana S. Santos, Luís Fonseca, Isabel M. O. Abrantes, Paula V. Morais

**Affiliations:** 1 IMAR – Marine and Environmental Research Center, University of Coimbra, Coimbra, Portugal; 2 Center for Neuroscience and Cell Biology, University of Coimbra, Coimbra, Portugal; 3 Department of Life Sciences, FCTUC, University of Coimbra, Coimbra, Portugal; CEA (Atomic and alternative energies commission), France

## Abstract

Bacteria associated with the nematode *Bursaphelenchus xylophilus*, a pathogen of trees and the causal agent of pine wilt disease (PWD) may play a role in the disease. In order to evaluate their role (positive or negative to the tree), strains isolated from the track of nematodes from infected *Pinus pinaster* trees were screened, *in vitro*, for their nematicidal potential. The bacterial products, from strains more active in killing nematodes, were screened in order to identify and characterize the nematicidal agent. Forty-seven strains were tested and, of these, 21 strains showed capacity to produce extracellular products with nematicidal activity. All *Burkholderia* strains were non-toxic. In contrast, all *Serratia* strains except one exhibited high toxicity. Nematodes incubated with *Serratia* strains showed, by SEM observation, deposits of bacteria on the nematode cuticle. The most nematicidal strain, *Serratia* sp. A88copa13, produced proteases in the supernatant. The use of selective inhibitors revealed that a serine protease with 70 kDa was majorly responsible for the toxicity of the supernatant. This extracellular serine protease is different phylogenetically, in size and biochemically from previously described proteases. Nematicidal assays revealed differences in nematicidal activity of the proteases to different species of *Bursaphelenchus*, suggesting its usefulness in a primary screen of the nematodes. This study offers the basis for further investigation of PWD and brings new insights on the role bacteria play in the defense of pine trees against *B. xylophilus*. Understanding all the factors involved is important in order to develop strategies to control *B. xylophilus* dispersion.

## Introduction

A large number of species belonging to different phylogenetic groups such as viruses, bacteria, fungi, insects and some invertebrates have been found capable of invading or preying on nematodes [1,2]. Nevertheless, chemical control is still the most commonly used method for managing nematode pests [3].

*Bursaphelenchus xylophilus*, the pine wood nematode (PWN), is one of the most concerning species, since it is a pathogen of coniferous trees and the causal agent of the pine wilt disease (PWD). *B. xylophilus* is native to North America where it is considered to cause little damage to trees. However, in regions where it was introduced, as for example Japan, China or Europe, it is one of the most destructive pests of forest and landscape pines. Understanding the infection process, and all the factors involved, in order to develop strategies to control *B. xylophilus* dispersions has been an objective in several continents (reviewed in Jones et al. [4]). 

During PWD development, it was observed that cell death in the host tree seems to occur in advance to the increase of nematode population. In order to find a justification for this observation, some authors considered the presence of phytotoxins, including 8-hydroxycarbotanacetone and 10-hydroxyverbenone, identified in nematode-infested plants, responsible for cell death in the host [5,6]. 

More recently, a potential role for bacterial symbionts in the disease process has been anticipated. Nevertheless, there are no data from USA or Europe to support the idea that *B. xylophilus* interact in symbiosis with bacteria to cause the disease.

A series of studies have shown that bacteria from various genera could be isolated associated with *B. xylophilus* [7–11]. The presence of bacteria on the nematode surface has been described [12], and a report on the observation of bacteria, by transmission electron microscopy, in the intestine of PWN was also performed [13]. Lately, a Gram negative bacterium, *Stenotrophomonas maltophilia*, isolated from soil, was described [14] to have nematotoxic activity against *B. xylophilus*, killing 65% of the nematodes after 24h of incubation. The analysis of the virulence factors revealed the presence of an extracellular serine protease of 28 kDa able to digest the nematodes cuticle. Among the hydrolytic enzymes, serine proteases have recently been shown to be very important in the penetration and digestion of nematodes by nematode-trapping fungi [15–18].

Bacteria associated with *B. xylophilus* and other nematodes isolated from *Pinus pinaster* trees with pine wilt disease [9] from recently nematode invaded areas (pine forest areas invaded in 2008), were diverse and belonged to different species according to the geographical area where the nematodes were isolated [9]. *P. pinaster* from these studied areas had also a diverse endophytic microbial community [19]. 

In order to evaluate the potential of these associated bacteria to produce negative effects on the nematodes, the isolates associated to the nematodes infecting *P. pinaster* from new invaded areas in Portugal [9] were screened for their potential in killing *B. xylophilus in vitro*. The bacterial strains, more active in killing nematodes, were selected and the bacterial extracellular products, produced during growth, were studied in order to determine their nematicidal activity. 

## Materials and Methods

### Ethics Statement

No specific permissions were required for sampling, since the study was supported by "Fundo Florestal Permanente" (Permanent Forest Fund), through a national project. The field studies did not involve endangered or protected species.

### Bacterial isolation

Bacteria strains used in this work were isolated in Proença et al. [9]. Briefly, *P. pinaster* trees were sampled in three different Portuguese areas affected by PWD: one located in southern Portugal, Setúbal District, between Alcácer do Sal and Grândola (Z) and two other areas in Central Portugal, Coimbra District, located in Malhada Velha, Arganil (M) and Avô, Oliveira do Hospital (A). The area between Alcácer do Sal and Grândola (Z) is affected with PWD since 1999 and the areas M and A are affected with PWD since 2008 [20]. The presence of nematodes was screened in all symptomatic trees. The bark and sapwood of infected trees were removed under sterile conditions, and the wood cut in ca. 2 cm chips. The wood pieces were placed in Petri dishes with R2A medium and incubated at 25°C, for three days. All bacterial colonies were isolated from the trails made by the nematodes on the medium. Isolated strains were preserved in LB medium with 15% glycerol at -80°C. 

### Screening and identification of bacterial strains with nematicidal activity

The bacterial strains, isolated under the conditions described above, were grown in Casamino Acids liquid medium (CAA) [21] at 180 rpm, during 24h until the end of the exponential phase, at 26°C. The suspensions obtained were centrifuged (20 min, 4°C, 4000 rpm), in order to drastically reduce the number of cells, without removing any growth product (filtration with 0.22 µm filter showed to affect nematicidal activity in some strains). To test the nematicidal activity, 500 μl of each bacteria supernatant, with less than 0.06 O.D._600nm_, were incubated with 75 (3 times 25) disinfected nematodes, for 48 hours at 26 °C. Nematodes were disinfected by sequential washes in sodium hypochlorite 0.1 % (one wash, 1 min at 4°C) and in 1 ml sterilized water (two washes, 3 min at 4°C), followed by centrifugation. Last water (100 μl) was inoculated on R2A for control of the disinfection efficiency.

The number of dead nematodes was assessed under the stereoscopic microscope. Nematodes were considered dead when linearized and not able to recover after being transferred to water. All tests were performed in triplicate and repeated at least two separate times. The controls - nematodes in CAA and nematodes in water - were incubated under the same conditions. 

The bacterial strains with a significant nematicidal activity in the above screening were selected and identified by 16S rRNA gene sequencing and comparison with sequences available in the EMBL/GenBank database, using BLAST network services, and with sequences in the EzTaxon identification service [22]. 

### Screening for extracellular proteases

To screen for the presence of proteases, all strains were grown in 50 ml of CAA at 26°C during 24h, centrifuged (20 min, 4°C, 4000 rpm) and the resulting supernatants were evaluated for the presence of proteins by SDS-PAGE using slab gels (1.5mm thick, 10% polyacrylamide) [23]. The proteins were stained with Coomassie Blue G-250. Additionally, the proteolytic activity of the supernatants was evaluated by zymography using 12.5% polyacrilamide gels containing 0.2% copolymerized gelatin. In parallel for comparison, a native gel without gelatin was also performed. Each supernatant (25 μl) was mixed, without boiling, with sample buffer (dilution 1:1 with 125 mM Tris-HCl, pH 6.8, containing 4% SDS and 20% glycerol) and applied to the gel. After electrophoresis, zymogram gels were renatured twice with 0.25% Triton-X-100, washed in distilled water and incubated overnight in activation buffer (Tris HCl 50 mM pH 8.0). Gels were stained with Coomassie Blue and proteolysis in zymograms was detected as clear zones in a blue background. The weight of the proteins was estimated with an appropriate marker (Precision Plus Protein Standard) for native gel analysis (Bio-Rad). 

Extracellular proteases of the strain A88copa13 were assessed from supernatants as described above. To purify the extracellular proteases with proteolytic activity, the purification process was initiated with a step of concentration of the collected supernatant trough Millipore Centricon® Centrifugal Filter Unit with a cut-off for 30,000 MW. The protein content was determined in the elute by Bradford assay (Bio-Rad Protein Assay, Bio-Rad). The proteins were purified by anionic exchange in FPLC separation. The crude protein extract was applied to a Hitrap Q column (1 ml) (Amersham, Switzerland) equilibrated in (buffer A) 50 mM Tris HCl (pH 8.0) buffer. The column elution was performed by a linear gradient 0-50% of (buffer B) 50 mM Tris HCl (pH 8.0) with 1 M NaCl for 20 minutes at 1 ml/ min flow rate. The eluted proteins were detected at UV 280 nm. Fractions were collected and assayed for protease and nematicidal activities.

To assess the protease activity, 200 μl of each fraction and 2 μl of each of the 12 different substrates indicated in Table 1 were added to 50 mM Tris HCl, pH 8.0. All substrates were at 10 mM concentration. The release of AMC was measured for 20 minutes at 37°C, using a microplate fluorometer SpectraMAX-GeminiEM (Molecular Devices, Sunnyvale, USA), at an excitation wavelength of 380 nm and an emission wavelength of 460 nm. The fractions with the higher proteolytic activity were tested for nematicidal activity as described below. The proteolytic inhibition profile using Ala-AMC as substrate was determined by adding to the protease activity assay different inhibitors (Table 1).

**Table 1:** Proteolytic inhibition profile of the supernatant from *Serratia* strain A88copa13 with Alanine-AMC substrate.

**Inhibitor**	**% remaining**
	**activity**
No inhibitor	100
Pefabloc SC (1mM)	12
1,10 Phenanthroline (10mM)	13
Amastatin (10µM)	43
TPCK (0.1mM)	48
TLCK (0.1mM)	51
E-64 (10µM)	57
Bestatin (10µM)	57
Aprotinin (0.08mM)	59
Α1-Antitrypsin (0.002mM)	59
chymostatin (0.1mM)	63
Pepstatin (1µM)	68
EDTA (10mM)	74
PMSF (1mM)	74
Mn^2+^ (1mM)	152
Mg^2+^ (1mM)	61
Ca^2+^ (1mM)	51
Zn^2+^ (1mM)	34

### MALDI-TOF identification of the proteases and gene amplification in strain A88copa13

The bands in native acrylamide gel, corresponding to proteins with proteolytic activity, were excised and placed into 1.5 mL microtubes, covered with water and sent to be sequenced by MALDI-TOF/TOF (MS / MS) at IPATIMUP, Porto, Portugal. Briefly, the samples were digested with trypsin, followed by the search of proteins from the mass spectrum of the peptides obtained (PMF). Peptide fragmentation spectra was obtained by peptide sequencing of MS / MS peaks with higher signal-noise. Peptides resulting from autolysis of trypsin were used as internal calibrants. Searches in databases of proteins and in the NCBInr Swiss-Prot/UniProtKB selections for taxonomic Bacteria and all entries were carried out.

The detection of the genes coding for the proteases in strain A88copa13 was done by PCR amplification with PCR reaction mix (50 μl) containing: reaction buffer (1.5 mM MgCl_2_, 50 mM KCl and 10 mM Tris-HCl, pH 8.3), 100 μM (each) deoxynucleoside triphosphates (Promega, Madison, Wisconsin, USA), 0.2 μM (each) primer and 1.5 U Taq polymerase (Sigma, St. Louis, Missouri, USA). For the Serralysin gene amplification the primers were 50KRT (5'-TTACACGATAAAGTCCGTGGCG-3') and 50KFT (5'-TGAGTGGAATCGAACCAATGCA-3'). Amplification of Serine protease gene was performed by three sets of primers 70KF1 (5'-GCATGYGTTATCYGTCGTTTC-3') and 70KR1 (5'-CAGTTCGCCCTTATTATAMGCT-3'), 70KF2 (5'-AGTTAAGCGTTTCCTCRAC-3') and 70KR2 (5'-GATGCTCGCCTCTTCGTTCAC-3'), 70KF3 (5'-GAAACGGCCAAACGCAGCGTG-3') and 70KR3 (5'-ATCGTYKCGATGRGGGCTGA-3'). The PCR was performed with 30 cycles: 1 min at 94°C, 1 min at 53°C, and 1 min 30 sec at 72°C. PCR products obtained were purified using the JET Quick PCR Purification Spin Kit (Genomed GmbH, Löhne, Germany) according to the manufacturer’s instructions and sequenced.

### Nematicidal activity of the proteases

The FPLC fractions, presenting higher protease activity, were tested for nematicidal activity against *B. xylophilus* (Bx-Portugal11AS) and other species of the genus *Bursaphelenchus*. The nematodes from the species *B. tusciae* (Bt-Italy), *B. mucronatus* (Bm-Portugal2) and *B. conicaudatus* (Bc-Japan) were used to evaluate the specificity of the proteases against the different species of nematodes, using the methodology described above. Inhibitors for the different classes of proteins were added to the FPLC fractions with protease activity and incubated during 20 min. Each mixture was then incubated with 25 nematodes, in triplicate, under the conditions described above, and the nematodes mortality was evaluated. Controls were performed by incubating nematodes with the protease inhibitors used, in order to evaluate their toxicity under the same conditions: 1 mM Pefabloc SC (Sigma), 10 mM EDTA (Sigma) and 10 mM 1, 10-Phenantrolin (Sigma Aldrish). 

### Scanning Electron Microscopy observations

PWNs subjected and not subjected to nematicidal tests were prefixed in 2% formaldehyde plus 2% glutaraldehyde in 0.1 M phosphate buffer (pH 7.2) for 12 h at 4°C. The nematodes were post-fixed with 2% osmium tetroxide for 30 min, and then dehydrated with serial ethanol treatments (30, 50, 70, 90, and 95%, and twice in 100% ethanol) for 30 min at each concentration. Then, the nematodes were dried by a critical point dryer (JCPD-5; JEOL, Tokyo) using liquid carbon dioxide, and sputter-coated with Au-Pd using a magnetron sputter (MSP-1S; Vacuum Device, Ibaraki, Japan). Scanning electron microscope (SEM) images were acquired using a VE-8800 (Keyence, Osaka, Japan).

### Statistical Analysis

The significance of the differences, observed between the different treatments with *Serratia* sp. A88copa13, was evaluated with analysis of variance (ANOVA), using Tukey's Multiple Comparison Test, considering: 1) the amount of NaCl in the supernatant, 2) the different fractions obtained after FPLC of the supernatant, and 3) different species of nematodes. The statistical analysis was performed with GraphPadPrism v5.0 for windows, GraphPad software, San Diego, California, USA, www.graphpad.com. All differences were considered to be statistically signiﬁcant for *P* < 0.05.

## Results

### Screening of the bacterial nematicidal activity

Forty-seven bacterial strains (Table 2) were evaluated for their ability to kill *B. xylophilus* nematodes. From these, 21 strains shown to have nematicidal activity (100% of nematodes dead after the incubation period). Five belonged to the species *Serratia plymuthica*, five to *Pseudomonas lutea*, three to *Serratia marcescens*, two to *Rhanella aquatilis* and one strain to each of the species *P. koreensis*, *P. constantinii*, *P. putida*, *P. moorei*, *Pantoea cypripedii* and *Curtobacterium pusillum* (Table 2). None of the *Burkholderia* strains had nematicidal activity (less than 30% of the nematodes killed during the assay). The lack of nematicidal activity was also observed in one strain of the genus *Yersinia* and in two *Erwinia* strains, and in one *P. putida* strain. 

**Table 2:** Evaluation of the nematicidal ability of the bacteria isolated associated with nematodes.

			**16S rRNA gene**							
**Families**	**Species identification**	**Strain**	**Accession number**	**Nematicidal**	**Siderophores^a^**	**Proteases^a^**	**Lipases^a^**			
**Classification**			**(DDBJ/EMBL/GenBank)**	**activity (%)**	**(CAS)**	**(skim milk)**	1	2	3	4
*Microbactereaceae*	*Curtobacterium pusillum*	**M24copaD2**	HQ538819	100	+	-	-	+	+	-
*Xanthomonadaceae*	*Luiteibacter rizhinovicinus*	**M24copaE1**	HQ538818	70-90	+	+	-	-	-	-
	*Cronobacter* sp.	**A37tronco2**	HQ538803	70-90	+	-	+	+	+	-
	*Cronobacter* sp.	**A37tronco5**	HQ538804	70-90	+	-	+	+	+	-
	*Erwinia* sp.	**B23tronco1**	HQ538806	70-90	+	-	-	+	-	-
	*Erwinia* sp.	**A12tronco1**	HQ538805	0-10	+	-	-	-	-	-
	*Erwinia* sp.	**A88tronco1**	HQ538807	0-10	+	-	-	-	-	-
	*Pantoea* sp.	**A37tronco4**	HQ538808	70-90	+	-	-	-	-	-
	*P. cypripedii*	**A37tronco3**	HQ538809	100	+	-	-	-	-	-
	*Yersinia intermedia*	**A88copa1**	HQ538815	30-50	+	-	-	+	+	-
	*Rhanella aquatilis*	**M72troncoB**	HQ538816	100	+	-	+	+	+	+
*Enterobactereaceae*	*R. aquatilis*	**M72troncoD**	HQ538817	100	+	-	-	+	-	-
	*Serratia plymuthica*	**M24tronco1**	HQ538810	100	-	-	-	-	-	-
	*S. plymuthica*	**M24tronco3**	HQ538811	100	+	-	-	-	-	-
	*S. plymuthica*	**M24tronco4**	HQ538812	100	+	-	-	-	-	-
	*S. plymuthica*	**M24tronco5**	HQ538813	100	+	-	-	-	-	-
	*S. plymuthica*	**M24tronco6**	HQ538814	100	+	-	-	-	-	-
	*S. marcescens*	**A37tronco1**	HQ538802	70-90	+	+	+	+	+	+
	*S. marcescens*	**A88copa5**	HQ538799	100	+	+	+	+	+	+
	*S.marcescens*	**A88copa7**	HQ538800	100	+	+	+	+	+	+
	*S.marcescens*	**A88copa13**	HQ538801	100	+	+	+	+	+	+
	*Burkholderia* sp.	**M47tronco8**	HQ538795	0-10	+	-	+	+	+	+
	*Burkholderia* sp.	**Z1S-T2**	FJ784703	0	-	-	+	+	+	-
*Burkholderiaceae*	*Burkholderia* sp.	**Z1S-T3**	FJ784709	0-10	+	-	+	-	+	+
	*Burkholderia glathei*	**M47tronco6**	HQ538796	10-30	+	-	+	+	+	+
	*B. fungorum*	**M72copaA**	HQ538798	0	+	-	+	+	+	+
	*B. sordidicola*	**M47copa4**	HQ538797	10-30	+	-	+	+	+	+
	*Pseudomonas* sp.	**M47copa1**	HQ538794	70-90	+	-	-	+	+	-
	*P. constantinii*	**A25tronco1**	HQ538793	100	+	+	+	+	+	+
	*P. koreensis*	**M47copa9**	HQ538786	100	+	-	-	+	+	-
	*P. koreensis*	**M47copaA**	HQ538787	70-90	+	-	-	-	-	-
	*P. gengeri*	**M72copaC**	HQ538789	70-90	-	-	-	-	-	-
	*P. lutea*	**M24copa1**	HQ538781	70-90	+	-	+	-	-	-
	*P. lutea*	**M24copa10**	HQ538776	70-90	+	-	+	+	+	-
	*P. lutea*	**M47copa3**	HQ538780	100	+	-	+	-	-	-
	*P. lutea*	**M47copa6**	HQ538778	70-90	+	-	+	-	-	-
	*P. lutea*	**M47copa11**	HQ538777	100	+	-	+	-	+	-
*Pseudomonadaceae*	*P. lutea*	**M47copa12**	HQ538783	70-90	+	-	-	+	-	-
	*P. lutea*	**M67copa5**	HQ538775	100	+	-	+	-	-	-
	*P. lutea*	**M67copa7**	HQ538782	100	+	-	+	-	-	-
	*P. lutea*	**M67copa9**	HQ538784	70-90	+	-	+	-	-	-
	*P. lutea*	**M24copaA**	HQ538779	100	+	-	+	+	-	-
	*P. lutea*	**M24copaE2**	HQ538785	70-90	+	-	-	-	+	-
	*P. moorei*	**M47tronco1**	HQ538790	100	+	-	-	-	-	-
	*P. putida*	**M67copa10**	HQ538791	100	+	-	-	+	-	-
	*P. putida*	**M68copaB**	HQ538792	70-90	-	-	-	-	-	-
	*P. putida*	**M72copaD**	HQ538788	10-30	+	-	-	-	-	-

Characterization of some catabolic properties and siderophores production that can be involved in the nematicidal activity. Lipase activity was tested in Tween 20 (1), Tween 40 (2), Tween 60 (3) and Tween 80 (4).

^a^Data obtained from Proença et al. 2010.

All strains showed ability to produce siderophores except 4 strains from three different genera (*Burkholderia*, *Pseudomonas* and *Serratia*), all with different nematicidal activities. Proteolitic activity in skim milk was detected in the supernatant of *P. constantinii*, *L. rizhinovicinus*, and of the 4 strains of the species *S. marcescens*, all able to kill more than 70% the nematodes during the assays. Only strains from *R. aquatillis* (one strain), *S. marcescens* (four strains) and *P. constantinii* (one strain) were able to degrade all the different lipids tested including Tween 80 (Table 2). All these strains were able to kill more than 70% of the nematodes in the nematicidal assay. 

**Figure 1:**
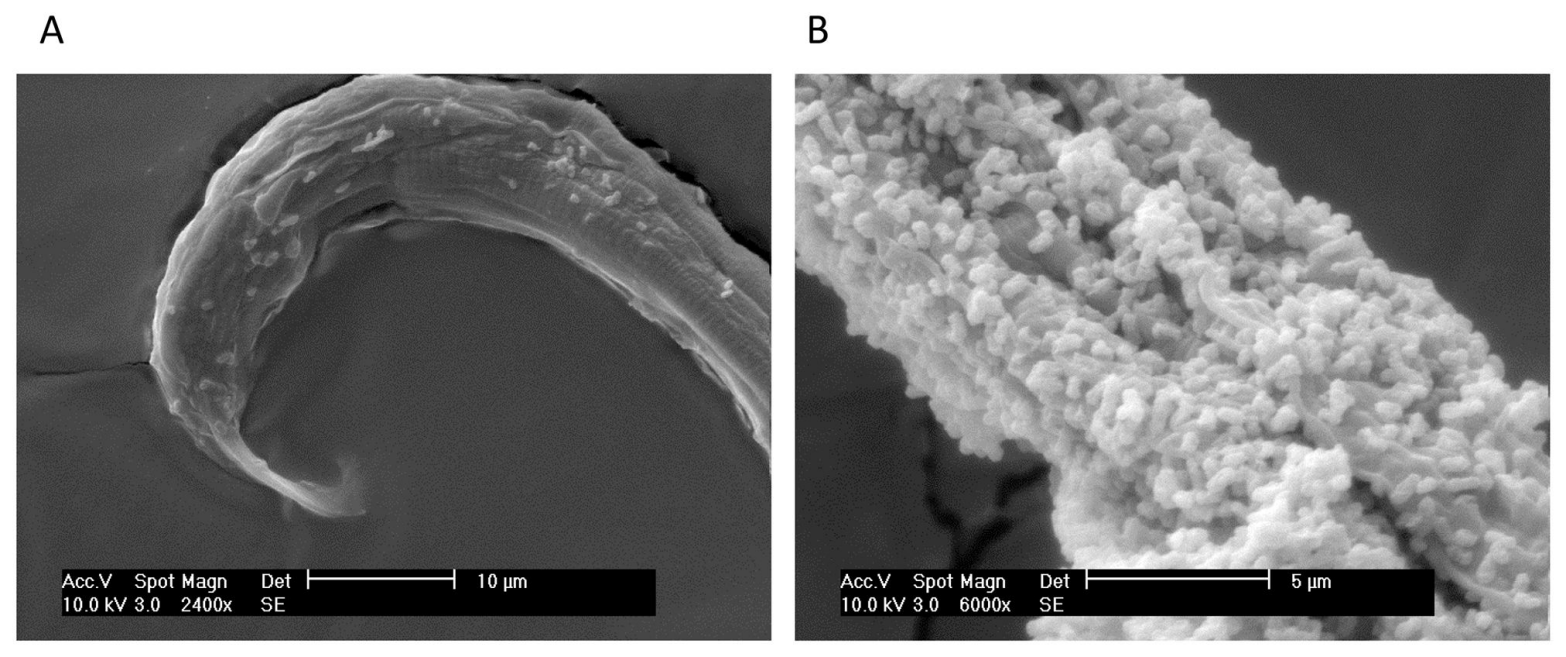
SEM images of deposits of *Serratia* strain A88copa13 on the cuticle of the nematode *Bursaphelenchus xylophilus*. Incubation after 24 h (A) and 48 h (B).

The observation by SEM of the result of bacterial activity on the nematodes showed bacteria cells accumulated on the nematode cuticle (Figure 1). The incubation of the nematodes with bacteria lead to the degradation of the nematodes, and only bacteria-covered empty cuticles were visible after 48h incubation.

### Proteases in the supernatants of nematicidal bacteria

**Figure 2:**
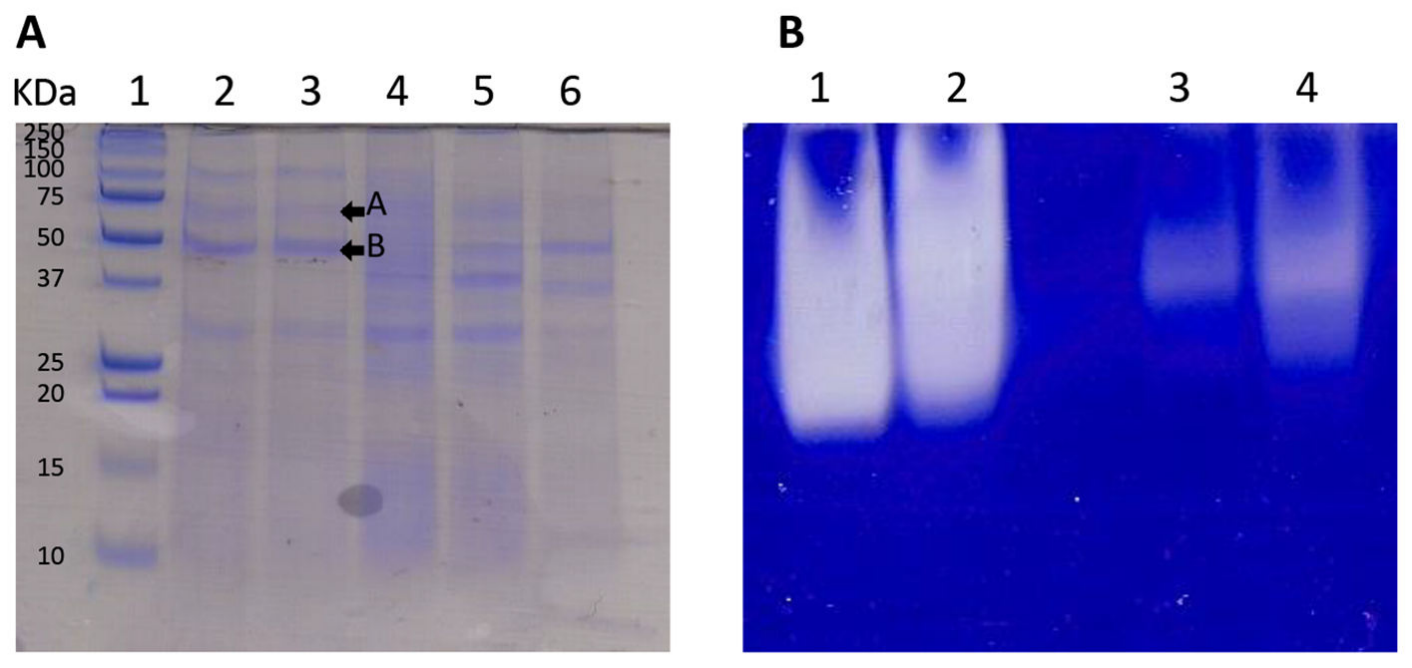
Proteins in the supernatants of nematicidal bacteria. (A) SDS-PAGE of extracellular proteins from *Serratia* strains A88copa13 (2 and 3) and A88copa5 (4–6), molecular weight marker (1). Arrows indicate the band of 70 kDa (A-serine protease) and the band of 50 kDa (B-metalloproteinase serralysin) removed for sequencing by MALDI-TOF/TOF (MS / MS). (B) Zymogram using 12.5% polyacrilamide gel containing 0.2% copolymerized gelatin of extracellular proteins of *Serratia* strains A88copa13 (1 and 2) and A88copa5 (3 and 4) showing differences in protease activity.

The strains with higher nematicidal activity were selected to be screened for the presence of extracellular substances and proteins with proteolytic capacity. The strains A25tronco1, A37tronco1, A88copa5, A88copa7 and A88copa13 produced soluble extracellular proteins with proteolytic activity when evaluated by zymography with gelatin. Strain *S. marcescens* A88copa13 was selected for further work, because it produced the proteins with highest activity in the zymogram gel (Figure 2).

### Identification of extracellular proteins with proteolytic activity

The production of extracellular proteins by strain A88copa13, under the growth conditions used, was independent of the presence of nematodes (added dead to the growth medium) during strain growth (data not show). During growth of strain A88copa13, aliquots of the supernatant were removed at different times and the nematicidal activity was evaluated. The nematicidal activity of the strain's supernatant increased during the exponential phase of the strain's growth and was maximal when the strain achieved 2.4 O.D._610nm_ (late exponential). Therefore, all assays were performed with aliquots removed from strain growth with 2.4 O.D._610nm_. The amount used to purify the protein fractions was standardized to 50 ml of supernatant. The protein content of the supernatant was 1.23±0.08 mg/ml, when quantified after centrifugation, and shown to be stable.

The toxicity assays were incubated for three days, and during this period the number of dead nematodes was counted each 24h. No increase in the number of dead nematodes was observed after 48h incubation. After this time, a brown color developed in the supernatant and the proteolytic activity ceased.

The presence and the number of proteins in the supernatant of the different nematicidal strains was evaluated by SDS-PAGE. The band from the supernatant of strain A88copa13 with 50 kDa (Figure 2) was identified by MS / MS, with 100% statistical significance (SwissProt database), as the extracellular metalloproteinase serralysin. The band with 70 kDa was identified, with 100% statistical significance (SwissProt database), as an extracellular serine protease. In strain A88copa13, the gene sequence obtained for the 70 kDa protease (JX667979) was 95% similar to the serine protease gene sequence of *S. marcescens* IFO-3046 (M13469). On the other hand, the 50 kDa band was coded by a gene (JX667978) which had 96% similarity with the *S. marcescens* strain ZhenJiang insecticidal protein gene (EU999787). 

The comparative analysis of the amino acid sequence obtained from nucleotide translation of the 70 kDa protease using BlastX showed an high homology (96%, 87%, 50% and 49%, respectively) with the serine proteases of the subtilisin-type serine proteases family from two strains of *S. marcescens*, (P09489, P29805) and with a smaller serine protease (60 kDa) from *Xenorhabdus bovienii* (YP_003468240) and *X. nematophila* (YP_003712486). Similarly, the comparative analysis of the amino acid sequence of the 50 kDa protease showed 99% homology with the zinc-dependent metalloproteinase, serralysin-like proteins from different strains of *S. marcescens* and *S. nematophila* (ACH90152, ABK55613, CAA39138 and ABZ81090).

### Effect of protease inhibitors and metal ions on the enzyme activity

To confirm biochemically to which group the proteases in *Serratia* sp. A88copa13 belong, their activity was measured testing the supernatant in the presence of different protease inhibitors and protein substrates (Tables 1 and 3). 

**Table 3:** Profile of proteolytic substrate specificity of the supernatant from strain *Serratia* sp. A88copa13.

**Substrate**	Activity*
	FU**/Min
Ala-AMC (10mM)	314.70
Boc-Val-Pro-Arg-AMC (10mM)	216.42
BzArg-AMC (10mM)	97.56
Ala-Ala-Phe-AMC (10mM)	70.32
Phe-AMC (10mM)	41.42
Arg-AMC (10mM)	24.64
Met-AMC (10mM)	23.56
Lys-AMC (10mM)	22.79
Suc-Ala-Pro-Ala-AMC (10mM)	19.60
Leu-AMC (10mM)	13.30
Gly-Pro-AMC (10mM)	9.55
Suc-Leu-Leu-Val-Thr-AMC (10mM)	0.33

* 50 ml of supernatant

** *FU* – Fluorescence Unity

The digestion of the fluorogenic substrates at pH 7.0 showed that Alanine-AMC and Boc-Val-Pro-Arg were the substrates that induced more activity (Table 3). These peptides were typical identified as metalloprotease (aminopeptidase) substrate and serine protease substrate, respectively.

To further characterize the extracellular proteases, enzymatic assays were performed with Alanine-AMC in the presence of class-specific protease inhibitors and divalent ions. In the presence of the substrate Alanine-AMC, the inhibitor Pefabloc SC (4-(2-Aminoethyl) benzenesulfonyl fluoride hydrochloride; AEBSF), when added in concentrations of 1 mmol/L, almost completely abolished the proteolytic activity of the supernatant, revealing a serine protease as a major active proteolytic enzymatic group (Table 1). The inhibitor 1,10-Phenanthroline, a chelating agent with high affinity to zinc, when added in concentrations of 10 mmol/L in the presence of Alanine-AMC as substrate, almost completely abolished the activity of the supernatant, also revealing an active metalloproteinase as a major proteolytic group, also. 

In addition, with Alanine-AMC as substrate, Aprotinin showed to have an effect on the proteolytic activity of the supernatant and inhibited the proteolytic activity in 41%. EDTA is a metal ions chelator, which can chelate Ca^2+^ ions, and is recognized as an inhibitor of metalloproteinases. The addition of EDTA to the supernatant partially inhibited (26%) its proteolytic activity. Aspartic protease inhibitor, Pepstatin A, showed a moderate effect in the supernatant, with 32% of inhibition. Alkali metal ions showed a moderate effect on the enzymatic activity of the supernatant. However, Zn^2+^ inhibited the supernatant activity (66%) while Mn^2+^ increased it (152%).

**Figure 3:**
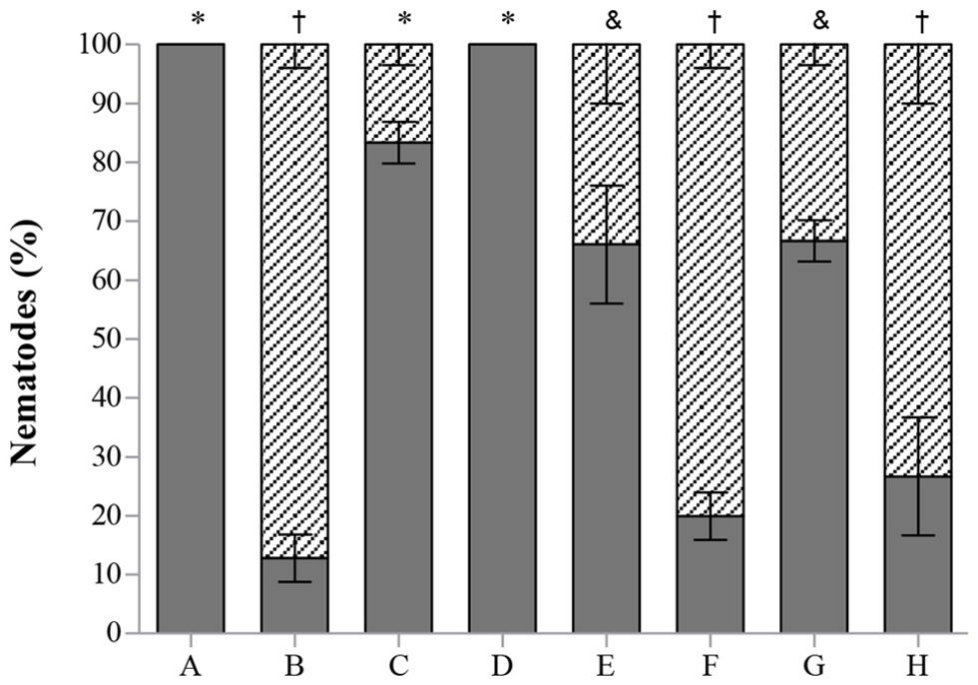
*Bursaphelenchus xylophilus* dead (stripes) and alive (grey) after the different *in* *vitro* treatments with *Serratia* strain A88copa13 (growth supernatant) and with proteases (FPLC fractions), after 24h incubation. The fractions with proteolytic activity contained 0.1M NaCl. A- H_2_O; B- *Serratia* strain A88copa13; C- *Serratia* strain A88copa13 + Pefabloc SC; D- H_2_O + Pefabloc SC; E- *Serratia* strain A88copa13 + EDTA; F- EDTA + H_2_O; G- FPLC protein fraction 9; H- FPLC protein fraction 19. Different symbols (*, †, &) above the bars indicate significantly different percentages of mortality (P < 0.05) between treatments.

The nematicidal activity of Pefabloc SC inhibitor was negative but EDTA and Phenantroline had a high nematicidal activity (P<0.05) (Figure 3). The high toxicity of the EDTA to the nematodes may suggest its action as a membrane permeabilizing agent, through the chelation of cations. When added to the supernatant, the amount of cations available is higher than in sterilized distilled water, and the EDTA free to chelate cations from membranes will be less, lowering its toxicity. On the other hand, the possibility of the strain to produce EDTA degrading proteins to the supernatant cannot be overruled [24].

### Nematicidal activity of FPLC fractions

**Figure 4:**
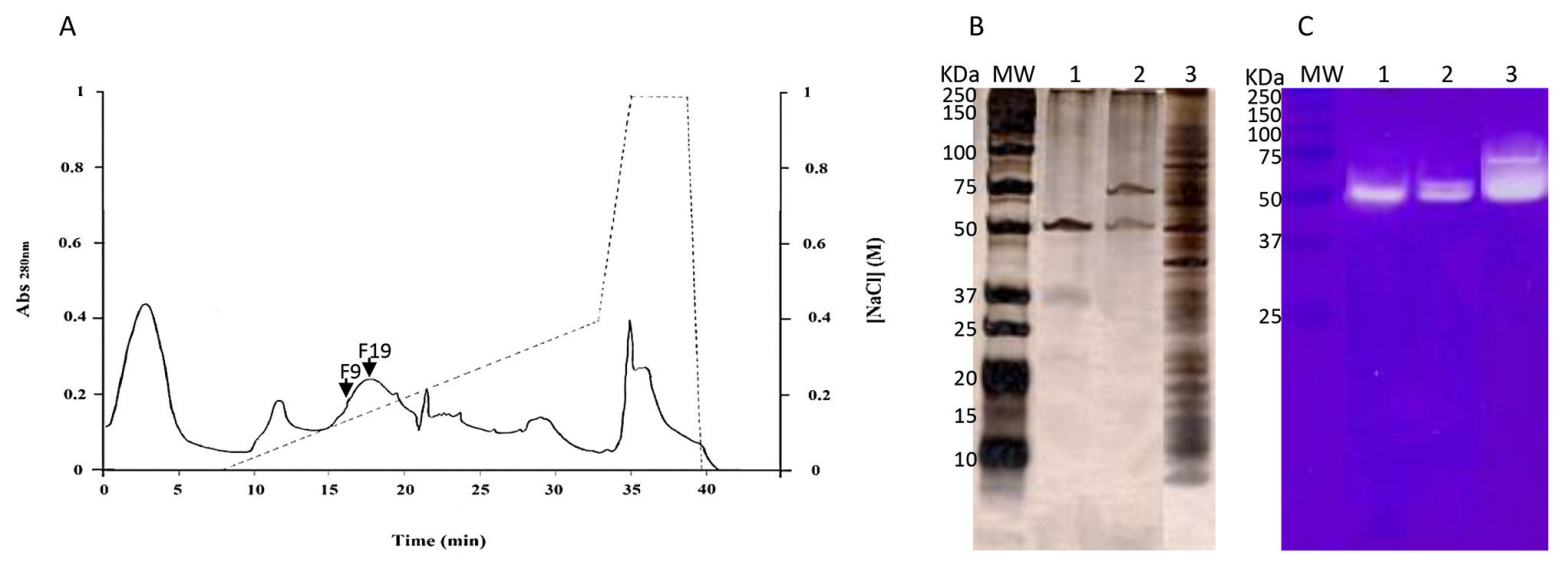
Purification and characterization of the proteases in the growth supernatant of strain *Serratia* A88copa13. (A) Anionic exchange chromatography on Hitrap Q column (1ml). Fractions 9 and 19 were eluted by using a linear gradient 0-50% of 50 mM Tris HCl (pH 8.0) with 1 M NaCl for 20 minutes at 1ml/ min flow rate. FPLC protein fraction 9 rich in metalloproteinase (1), FPLC protein fraction 19 rich in metalloproteinase and serine protease (2), A88copa13 supernatant (3), and molecular weight (MW) evaluated by SDS-PAGE silver stained (B) and by zymography using 12.5% polyacrilamide gel containing 0.2% copolymerized gelatin (C).

**Figure 5:**
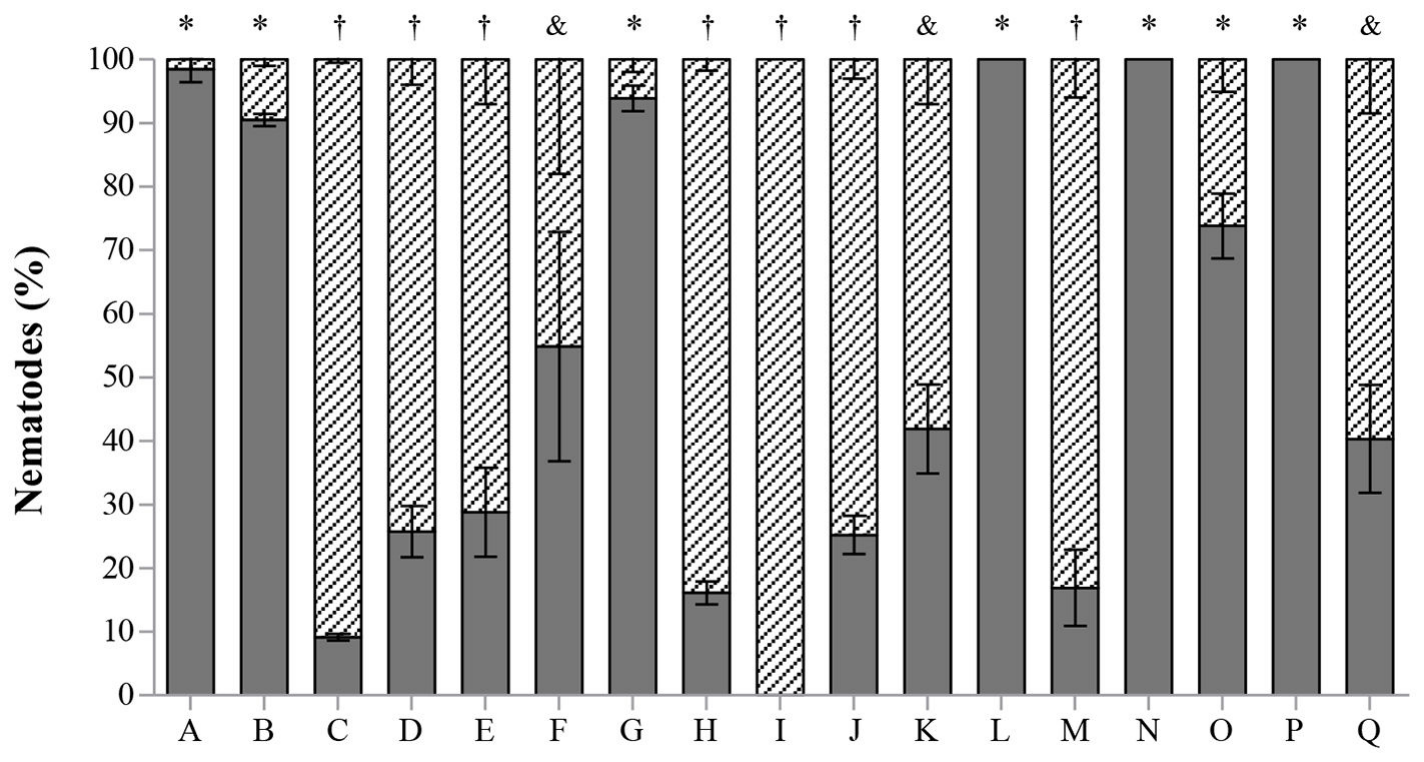
*Bursaphelenchus xylophilus* dead (stripes) and alive (grey) after the different *in* *vitro* treatments with *Serratia* strain A88copa13 (growth supernatant) and with proteases (FPLC fractions), after 48 h incubation. The fractions with proteolytic activity contained 0.1M NaCl. (A) H_2_O + Bx-Portugal11AS; (B) FPLC fraction containing the proteins that did not bound to the column + Bx-Portugal11AS; (C) Tris-HCl + (1M) NaCl + Bx-Portugal11AS; (D) Tris-HCl + (0.7M) NaCl + Bx-Portugal11AS; (E) Tris-HCl + (0.5M) NaCl + Bx-Portugal11AS; (F) Tris-HCl + (0.3M) NaCl + Bx-Portugal11AS; (G) Tris-HCl + (0.1M) NaCl + Bx-Portugal11AS; (H) FPLC fraction 19 + Bx-Portugal11AS; (I) *Serratia* strain A88copa13 (growth supernatant); (J) FPLC fraction 19 (75%) diluted with H_2_O; (K) FPLC fraction 19 (50%) diluted with H_2_O; (L) H_2_O + Bt-Italy; (M) FPLC fraction 19 + Bt-Italy; (N) H_2_O + Bc-Japan; (O) FPLC fraction 19 + Bc-Japan; (P) H_2_O + Bm-Portugal2; (Q) FPLC fraction 19 + Bm-Portugal2. Results from triplicate assays. Different symbols (*, †, &) above the bars indicate significantly different percentages of mortality (P < 0.05) between treatments.

Fractions of the supernatant separated by FPLC were tested for proteolytic activity and for their nematicidal activity (Figure 4A). A complete purification of the 2 proteases evaluated by SDS-PAGE and zymography was not achieved (Figure 4B). The FPLC fractions with proteolytic activity contained 0.1 M NaCl. Controls were performed in order to check the salinity effect on nematodes mortality. A control with the same salinity as the fractions tested (0.1 M NaCl), caused mortality similar to that observed with water (Figure 5). Statistical analysis showed that treatments with water (A, L, N, P), with proteins that did not bound to the FPLC column (B) and with buffer containing 0.1 M NaCl (G) did not induced nematodes mortality (P>0.05). The toxicity assays revealed that only two peaks showed nematicidal activity statistically comparable to the whole extract (other treatments with P<0.05). The FPLC fractions 9 and 19 showed the highest toxicity, although with significant statistical differences (P<0.05) in consequence, most probably, of the presence of an additional serine protease in fraction 19 when compared with fraction 9. The activity measured by the hydrolysis of substrates and by the inhibition of the activity, determined in the presence of the specific inhibitors of each protein, demonstrated that the serine protease had the highest nematicidal activity. After 24 h incubation, 73% mortality was observed when nematodes were incubated with fraction 19 (data not shown). Extending incubation increased *B. xylophilus* mortality to 83% (Figure 5). Dilution of these fractions lead to a decrease in toxicity. The nematicidal activity of fraction 19 after 24 h incubation was also observed against nematodes isolates of the species *B. tusciae* (Bt-Italy) (81%), *B. conicaudatus* (Bc-Japan) (13%) and also *B. mucronatus* (Bm-Portugal2) (10%). After 48h incubation, the nematicidal activity of this fraction increased significantly against Bm-Portugal2 and Bt-Italy (P<0.05) (Figure 5). Fraction 19 maintained the nematicidal activity against Bx-Portugal11AS until 72h of incubation but lost the activity when the incubation was prolonged (data not shown).

## Discussion

Several studies have demonstrated that under natural conditions, *B. xylophilus* can carry bacteria [7,9,25,26]. Some authors are convinced that those bacteria might play an important role in PWN pathogenicity [25,27] although the role of the bacteria carried by the nematode in PWD is not clear. The literature (reviewed in 28) supports the idea that bacteria isolated from nematodes from PWD trees, interact in symbiosis with the nematode to cause the disease but also points to the fact that there is no data from USA or Europe to support that idea. Therefore, considering that, microorganisms can produce a wide range of secondary metabolites, the objective of this work was to investigate the bacteria carried by the PWN for their potential nematicidal properties. 

The nematicidal ability was analyzed for 47 strains isolated associated to nematodes from trees with PWD. Only seven strains did not show toxicity against *B. xylophilus*, and *Burkholderia* was the only genus with all strains being non-toxic to the nematodes. Strains from the genus *Burkholderia* include well known human and plant pathogenic species (*B. gladioli*) but also some environmentally-important species such as *B. xenovorans* (chlororganic pesticides and polychlorinated biphenyls degrader) and some plant growth-promoting bacteria as *B. phytofirmans* [29]. However, the role *in vivo* of the strains of genus *Burkholderia* in *P. pinaster* with PWD was not evaluated. All strains of the genus *Pseudomonas*, except one strain of *P. putida*, showed toxicity against nematodes. The strains of the genus *Pseudomonas* are pointed by several authors as mutualistic with *B. xylophilus*, producing phytotoxins, and co-involved in PWD development [7,26,30]. In this study, the nematicidal activity of the bacterial extracellular products was demonstrated but we cannot overrule their toxicity to plants. The genus *Serratia* included the strains most toxic to the nematodes: all except one strain were able to kill 100% of the nematodes in 24h incubation. The nematicidal activity of these strains is higher than the nematicidal activity of the previously described nematicidal strain *Stenotrophomonas maltophilia*, which was able to kill 65% of the nematodes after 24h incubation [14]. Strains from genus *Serratia* were also isolated associated with *B. xylophilus* JJ2 of *P. densiflora* collected in Korea [31]. The strains of the genus *Serratia*, evaluated in this work, belonged to the species *S. plymuthica*, considered non-pathogenic, and to the species *S. marcescens*, considered pathogenic to humans. *S. plymuthica* is a ubiquitous bacterium that has been preferentially recovered from rhizospheres all over the world, both as a free-living and as an endophytic organism. This species acts by antibiosis, by the production of lytic enzymes, by competition for nutrients and iron, by secretion of siderophores, and by induction of plant defense mechanisms [32]. The strains of *S. plymutica* tested in this work produced proteases and siderophores but not lipases, and toxicity against the PWN was demonstrated *in vitro*. The draft genome sequence of *Serratia* sp. M24T3 (*S. plymuthica* – like) showed multiple genes potentially involved in virulence and nematotoxity [33] as the genes coding for colicin V and bacteriocin biosynthesis, as well as a set of genes typical for plant niche adaptation as, for example, acetoin (diacetyl) reductase that could be involved in plant protection against fungal and bacterial infections [33]. All strains from the species *S. marcescens*, differently from *S. plymutica* strains, produced lipases active on different polysorbates. This species was already reported as pathogenic to the pine sawyer beetle *Monochamus* [34]. 

In the process of enrichment of the proteases, aliquots were tested for proteolytic activity and for their nematicidal activity. The toxicity assays revealed that only two FPLC fractions (9 and 19) exhibited nematicidal activity comparable to the whole extract. The results from SDS-PAGE and enzymatic activity showed that these fractions included different concentrations of the two identified proteases (serine protease and serralysin). The use of selective inhibitors to serine proteases or to metalloproteinases demonstrated that the serine protease was the principal responsible for the toxicity on the PWN. 

The production of serralysin-like proteases has been detected in several *Serratia* strains related to insects [35]. The metalloproteinase is also produced by the entomopathogens bacteria from the genus *Xenorhabdus* and by *Photorhabdus luminescens*, both symbiotic with their nematodes [36]. In our work, the selective inhibition of serralysin did not reduce the nematotoxicity of the supernatant of strain *Serratia* A88copa13, which suggests that serralysin is not the nematotoxic protease. This is in agreement to the fact that nematode-symbiotic entomopathogenic bacteria also produce serralysin-like proteases.

Hydrolytic enzymes produced by Gram positive bacteria have been identified to be involved in the degradation of nematode components [37–39]. Alkaline serine proteases have been reported to be produced by *Brevibacillus laterosporus* strain G4 (30 kDa designated BLG4) and from *Bacillus nematocida* (28 kDa) [39,40] although both proteases were not the only virulence factor responsible for the nematicidal activities in these bacteria. Analysis of the results on nematode toxicity demonstrated that an extracellular serine protease was an important factor on strain A88copa13 virulence against the nematode *B. xylophilus*. The protease was also active against other nematodes of the genus *Bursaphelenchus*. However, considering the results after 24h, the nematicidal activity of the protease seems to be useful in the discrimination of the nematodes of the species *B. xylophilus* (100% of mortality with the supernatant) from the species of *B. mucronatus* (10% mortality)*. B. mucronatus* is morphologically very similar to *B. xylophilus* and, although it infects the trees, it is not pathogenic to pine trees. This is according to previous studies showing that surface coat proteins, probably crucial in the nematode modulating or evading the host immune response, were different between virulent PWN and the avirulent *B. mucronatus* (reviewed in 41). This serine protease is different, phylogenetically, in size and biochemically, from previously described proteases with nematotoxic activity but may play a similar role in infection against nematodes. 

Therefore, this study offers a basis for further investigation of the mechanism of PWD caused by PWN and brings new insights on the role bacteria can play on defense against *B. xylophilus* in the maritime pine trees. The diversity of roles that can be assumed by the same bacterial species, as *Serratia marcescens*, goes from plant grow promoting to phytopathogen through the acquisition of genetic mobile elements or environmental stimuli [42,43]. Understanding all the involved factors is important in order to develop strategies to control *B. xylophilus* dispersions.
